# A Population-Specific Major Allele Reference Genome From The United Arab Emirates Population

**DOI:** 10.3389/fgene.2021.660428

**Published:** 2021-04-23

**Authors:** Gihan Daw Elbait, Andreas Henschel, Guan K. Tay, Habiba S. Al Safar

**Affiliations:** ^1^Center for Biotechnology, Khalifa University of Science and Technology, Abu Dhabi, United Arab Emirates; ^2^Department of Electrical Engineering and Computer Science, Khalifa University of Science and Technology, Abu Dhabi, United Arab Emirates; ^3^Department of Biomedical Engineering, Khalifa University of Science and Technology, Abu Dhabi, United Arab Emirates; ^4^Division of Psychiatry, Faculty of Health and Medical Sciences, The University of Western Australia, Crawley, WA, Australia; ^5^School of Medical and Health Sciences, Edith Cowan University, Joondalup, WA, Australia; ^6^Department of Genetics and Molecular Biology, College of Medicine and Health Sciences, Khalifa University of Science and Technology, Abu Dhabi, United Arab Emirates

**Keywords:** UAE reference genome, next generation sequencing, structural variants, population representative sampling, population genetics, Arab genome, reference genome

## Abstract

The ethnic composition of the population of a country contributes to the uniqueness of each national DNA sequencing project and, ideally, individual reference genomes are required to reduce the confounding nature of ethnic bias. This work represents a representative Whole Genome Sequencing effort of an understudied population. Specifically, high coverage consensus sequences from 120 whole genomes and 33 whole exomes were used to construct the first ever population specific major allele reference genome for the United Arab Emirates (UAE). When this was applied and compared to the archetype hg19 reference, assembly of local Emirati genomes was reduced by ∼19% (i.e., some 1 million fewer calls). In compiling the United Arab Emirates Reference Genome (UAERG), sets of annotated 23,038,090 short (novel: 1,790,171) and 137,713 structural (novel: 8,462) variants; their allele frequencies (AFs) and distribution across the genome were identified. Population-specific genetic characteristics including loss-of-function variants, admixture, and ancestral haplogroup distribution were identified and reported here. We also detect a strong correlation between *F*_ST_ and admixture components in the UAE. This baseline study was conceived to establish a high-quality reference genome and a genetic variations resource to enable the development of regional population specific initiatives and thus inform the application of population studies and precision medicine in the UAE.

## Introduction

The lack of diversity in genome sequencing projects and Genome Wide Association Studies (GWAS) have led to a disproportionate representation of ethnicities in DNA sequence repositories. In non-European populations, individual whole genome sequencing efforts have been completed for the Yoruba-Nigerian (Bentley et al., 2008), Chinese (Wang et al., 2008), Korean (Kim et al., 2009; Cho et al., 2016; Seo et al., 2016), Japanese (Fujimoto et al., 2010), and Indian (Gupta et al., 2012; Almal et al., 2019) populations. Within the Arabian Peninsula, Qatar (Fakhro et al., 2016), Kuwait (John et al., 2015; Thareja et al., 2015), Saudi Arabia (Ibrahim Alabdulkareem et al., 2015), and most recently the genomes of the United Arab Emirates (UAE) (AlSafar et al., 2019; Daw Elbait et al., 2020) have been described. The significant reduction in the costs of DNA sequencing has enabled the upscaling of sequencing projects by several orders of magnitude. This has led to a proliferation of a number of national genome projects including the UK-100K project (Genomics England, 2019), the genome of the Netherlands using 750 Dutch genomes (Boomsma et al., 2014), and the 100 Southeast Asian Malays (Wong et al., 2013) project, among others. These large scales are necessary to provide the necessary statistical power to assess associations with genetic diseases as well as for identifying genetic diversity of a population [i.e., to detect rare and common variants and their population specific allele frequencies (AFs)].

However, despite all these efforts, genome science is failing in considering the diversity of the humans (Popejoy and Fullerton, 2016). For instance, the combined amount of all non-European samples in the GWAS catalog is only around 19% (Mills and Rahal, 2019). As a consequence, biomarkers in individuals of Asian and African descent could be potentially misclassified due to inaccurate statistical information on each variant (Petrovski and Goldstein, 2016). The situation is particularly dire for populations of the Middle East and Emirati people, who are represented by only 0.08 and 0.05% of samples in the GWAS catalog, respectively (Popejoy and Fullerton, 2016). Efforts to study the Arabian population genetics are recent, e.g., (Almarri et al., 2020; Tay et al., 2020), and their main focus has been directed toward genomic history and admixture events in the Middle East. However, to the best of our knowledge, no dedicated constructions of UAE population specific reference genomes have been conducted to date.

It is common practice to map sequences to an existing reference genome to map genetic variants. To date, the two versions of the human reference genome, namely the NCBI GRCh37 (hg19) and GRCh38 (Lander et al., 2001; NCBI, 2009), have been primarily relied upon. These reference genomes were constructed from a few individuals of mainly Caucasian backgrounds. A number of studies have revealed the limitation of these reference genomes when used for the identification of genetic variations that are population-specific (Hugo Pan-Asian Snp Consortium Abdulla et al., 2009; Li et al., 2010; Rosenfeld et al., 2012; Lu and Xu, 2013; Popejoy and Fullerton, 2016; Levy-Sakin et al., 2019; Sherman et al., 2019) which are not from the ethnic groups that are used to construct the reference genome, such as populations of the Arabian Peninsula. This is especially true among highly admixed and understudied populations such as those of the Arabian Peninsula. In the re-sequencing efforts of these groups, the suitable analyses of DNA reads will strongly depend on the suitability of the reference genomes that are used, to uncover the true nature of the genetic heritage of these populations.

Reference genomes will only represent the major variant of the individuals that were used to construct that reference. The low number of reference samples as well as the selection bias impacts variant mapping efforts, and thus subsequent genome analysis steps negatively. As a result, many national genome initiatives have tried to remedy the ethnic bias of the reference genome by producing population-specific reference genomes (Stark et al., 2019). For example, the Danish reference genome was constructed from sequencing and “*de novo*” assembly of 150 genomes of Danes (Maretty et al., 2017), the Qatari genome from 1,005 individuals from Qatar (88 whole genomes, 917 exomes) (Fakhro et al., 2016), and the Vietnamese reference from 50 genomes collected from the 1,000 genome project (Thanh et al., 2015).

The underrepresentation of genomes from the people of the UAE in global genome databases (Popejoy and Fullerton, 2016) makes analysis problematic. The information gleaned using genome references and AFs from other populations will impact negatively on the nation’s desire to implement precision medicine tools and individualized therapeutic strategies. This challenge led to the inception of the 1,000 UAE genome project (Al-Ali et al., 2018), an initiative with the goal to establish a high quality UAE specific Reference Genome (UAERG) that accounts for the unique and diverse genetic information of the UAE population. The knowledge generated from the analysis of the reference genome would support the accurate classification of genome variants and subsequently the development of personalized medicine strategies which are expected to contribute to improvements in the local healthcare system. In addition, the availability of a representative reference panel of genomes is likely to improve haplotype imputation accuracy of genotype arrays and hence increases their power in GWAS.

This study has established a baseline of the highest quality for future genome sequencing efforts in the UAE. A comprehensive catalog of short and structural variants (SVs) in particular novel variants found in the UAE population has been prepared and listed herewithin. These are fundamental to our understanding of human genetic variations in the UAE, in the context of the people who reside in the Middle East as well as global populations. This contribution is also a timely effort to counter the disproportionate representation of the genetic variation of Middle Eastern representatives, which if unaddressed, will lead to healthcare inequality in the application of precision medicine.

## Materials and Methods

### Sampling and DNA Extraction

A total of 1,028 UAE nationals were recruited for this study as part of the 1,000 Arab genome project (Al-Ali et al., 2018). One thousand of the samples were genotyped using the Illumina OmniExome genotype arrays (San Diego, CA, United States). Of these samples, 129 samples – 125 for the UAE genome reference construction and four for testing- were selected as the most representative individuals among the sampled set of the population (see section “Selection of Unrelated Samples and Admixture Calculation”). These 129 samples were sequenced using whole genome sequencing (WGS) and another 33 samples were sequenced using whole exome sequencing (WES) using Illumina paired-end sequencing technology.

All sequenced subjects provided their written informed consent. This study has been approved by the Institutional Ethics Committee of Mafraq Hospital in Abu Dhabi (MAF-REC_07). The inclusion criteria of the studied subjects were as follows: UAE nationals, >18 years old, capable to understand their contribution to the study, and the ability to provide informed consent. Saliva samples were collected from all subjects using the Oragene OGR 500 kit. The prepIT^®^L2P system was used to extract genomic DNA (gDNA) from the saliva samples. All experiments were performed in accordance with relevant guidelines and regulations.

### Library Preparation

Libraries for each individual were prepared from the cleaned and sheared gDNA using the protocol provided and recommended by the manufacturer of the Illumina TruSeq^®^ DNA PCR Free Library Prep kit (Illumina Inc., San Diego, CA, United States) and TruSeq Exome Library Prep kit in case of WES. The indexed paired-end libraries were then quantified using the Denovix DS 11 FX Fluorometer and sizes were confirmed using the Advanced Analytical Fragment Analyzer (Advanced Analytical Technologies Inc. where). The libraries were then sequenced using the Illumina Platform for paired-end WGS.

### Selection of Unrelated Samples and Admixture Calculation

For the selection of the most representative subjects among the recruited individuals from the UAE population, a phylogenetic tree from the genotype arrays of the 1,000 samples, each holding 2.3M variants after cleaning was constructed. The phylogenetic tree was generated using the identity-by-state distance measure from PLINK for creating the distance matrix and BioPython’s Phylo module to construct a neighbor joining tree. We select every eighth sample from the ordered list of tree leaves, to ensure that representatives were picked across the entire span of the phylogenetic tree. Furthermore, the KING (Manichaikul et al., 2010) tool was used to test for inferred relationships among the selected sample. The KING tool takes as input the. *bed* file compiled by PLINK from the genotype arrays.

**FIGURE 1:**
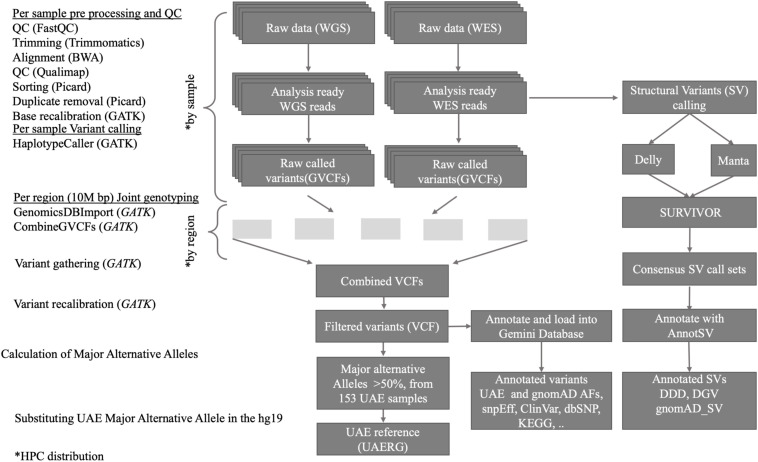
Overview of the data processing workflow, resources, and tools used for the joint short and structural variant calling and reference genome construction from the 153 UAE samples.

As outlined in Figure 1, the joint variant calling workflow is designed to run on our in-house high performance computing (HPC) center which comprises 92 compute nodes, each with 24 cores and 256 GB of memory, featuring the IBM LSF queuing system for parallel job processing. The workflow started with quality control (QC) processes that are applied to the raw data. Firstly, the FastQC tool (Andrews, 2010) was used, and by scanning the result files, only samples that passed the QC are used in the downstream analysis. The FastQC results also guided the parameter selection for the subsequent trimming step, where the Trimmomatic tool (Bolger et al., 2014), which removes low quality and short reads, was used. The Admixture calculation is described in (Tay et al., 2020).

### Variant Calling and Joint Genotyping Workflow

The raw reads from each of the 162 UAE nationals were mapped in parallel against the standard reference genome hg19 (Lander et al., 2001) using BWA-MEM v0.7.12 (Li and Durbin, 2010). The mapping quality and mean coverage per sample were calculated using Qualimap v2.2.1 (Garcia-Alcalde et al., 2012), where five genomic samples with coverage <10X were discarded, thus retaining 157 samples. Then, for each sample, the duplicate reads were marked and removed and the resulting BAM files were sorted by applying Picard v2.9.4 tools (Picard toolkit, 2018). The output files – one BAM file per sample – contain the analysis-ready reads for the subsequent processes.

Variants were called using the Genome Analysis Toolkit (GATK) version 4.0.6.0 Genomic Variant Call Format (GVCF) workflow (McKenna et al., 2010; DePristo et al., 2011). Variants from all samples were called individually using the GATK HaplotypeCaller with the *– ERC GVCF* parameter to produce GVCFs files. GVCF files were beneficial for joint genotype calling in the downstream analysis as they maintain sequencing depth information for non-variant loci which then facilitates the distinction of variant, reference, and no-call states for any site of interest.

The GVCF workflow enables us to operate on a population-wide scale by performing joint genotype calling from a cohort of multiple samples, thus improving variant calls in terms of accuracy. It inherits the advantages of classic joint variant calling over single and batch calling while being computationally efficient. The latter is owed to the fact that GATK’s Haplotype caller in GVCF mode can be run individually, and thus in parallel, on all samples (as shown in Figure 1, upper part). Subsequently, joint genotype calling on all 120 genomes and 33 exomes simultaneously were then performed. GATK’s new features Genomics DB Import and Genotype GVCFs were utilized. These steps were computationally expensive and were facilitated by parallel processing of GVCFs files split into 10M base pair sized regions (see Figure 1).

The Genotype GVCF step produces multiple VCF files that are then combined into one file that contains the integrated variants from all samples. The resulting VCF file is then subjected to filtering using the GATK VQSR which produces high-quality variants [single nucleotide polymorphisms (SNPs) and INDELs] that passed the VQSR filter.

### United Arab Emirates Reference Genome Construction and Testing

To identify the major AFs within the UAE samples with reference to the hg19, the AF for each SNP and INDEL’s positions were calculated. Then the positions where the reference alleles were different from the majority alleles on the UAE genomes were identified. Finally, to construct the UAERG, the hg19 reference genome was modified at these positions by replacing at each site in the hg19 with the major allele in UAE making it the new reference allele in the UAERG.

To assess the advantages of using the UAERG over the hg19 reference for variant detection in the UAE individuals, four extra whole genome sequenced samples were selected (samples not used in the reference genome construction). The variant calling pipeline was run using the raw data of each of the four samples where the mapping step was performed twice, one time against the standard genome (hg19) and another against the newly constructed reference genome (UAERG), for the four samples. The process resulted in two sets of called variants that were evaluated by calculating the difference of called variants from the two reference genomes.

The Python scripts for the reference genome construction have been made available at https://github.com/henschellab/ReferenceGenome. The repository also contains a reference genome construction script that works in parallel (by chromosome) on the previously mentioned HPC infrastructure.

The study by Fakhro et al. (2016) generated a similar dataset for 1,005 individuals from Qatar (88 genomes, 917 exomes). Each SNP in the UAE dataset was checked against the variant calling file from the Qatar dataset. Due to the nature of the dataset, the comparison to the UAE SNPs data was limited to biallelic SNPs (see script overlap.py).

### Annotation of Variants

In an effort to facilitate downstream variant analysis, the variant toolset, Vt (Tan et al., 2015) was used for decomposing (multi allelic variants) and normalizing the final variants call set (vcf file). The GEMINI tool was then used to annotate each variant by integrating several clinical and functional genome annotations including vcftools (Danecek et al., 2011), dbSNP, ClinVar (Landrum et al., 2018), snpEff (Cingolani et al., 2012), KEGG (Kanehisa and Goto, 2000), and gnomAD’s exons AFs. We additionally added the gnomAD’s genomes AFs to the database.

### Characterization of the UAE Ancestry Using Y and Mitochondrial DNA Haplogroups

The mitochondrial DNA (mtDNA) variants were extracted from vcf files generated from all samples. The resulting mtDNA vcf files were then lifted over to the revised Cambridge Reference Sequence of human mitochondrial DNA (rCRS) (Andrews et al., 1999) using the PICARD “LiftoverVcf” tool before using Haplogrep (Weissensteiner et al., 2016) to assign the respective mitochondrial haplogroups.

As for the identification of the Y-DNA haplogroups, the Y chromosome was extracted from the vcf files for each male sample using the bcftools. The Yhaplo (Poznik, 2016) python module yhaplo.callHaplogroups was then used to detect the haplogroups from the provided vcf files.

### Structural Variant Calculation

The SVs for the 120 WGS samples were generated using Manta (Chen et al., 2016) and Delly (Rausch et al., 2012) joint genotyping germline SV calling workflows parallelized on our in-house HPC. For consensus SV call sets from the results of the two callers, the SURVIVOR (Jeffares et al., 2017) tool was used to merge across SV callers and individuals and generate a union call set and an intersection call set, for which the SVs frequency was calculated. The tool AnnotSV (Geoffroy et al., 2018), an integrated tool for structural variations annotation; including annotations from DDD and DGV, and gnomAD_SV; was used to annotate the SV calls as illustrated in Figure 1.

### Visualization and Pathway Enrichment Analysis

Visual representation of the spatial variability of SNVs and SVs across the UAE genomes has been generated using Circos (Krzywinski et al., 2009). Furthermore, the variants of the highest peaks of the visualization, has been selected to perform Reactome Pathways (Croft et al., 2011) Enrichment analysis using SNPnexus (Dayem Ullah et al., 2012).

## Results

Sequences from 153 (120 WGS and 33 WES) UAE nationals were used to comprehensively describe the genetic make-up of the local population. Observed genotypes and AFs were defined. The collective variant calls were used to construct the UAERG. The suitability of such a bespoke reference genome was assessed by calling variants on the four test samples. One motivation for building the UAERG was to exclude variant calls that are predominant in the UAE population and are thus very unlikely to be disease relevant. In addition, several informative statistics on genome variants and population metrics were calculated.

### Sample Selection and Information on Subjects and Alignment Statistics

**FIGURE 2:**
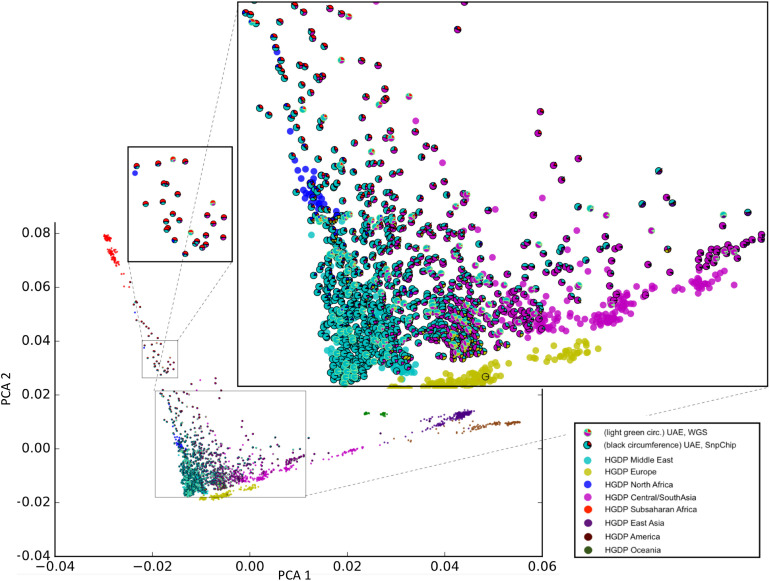
Principal component analysis (PCA)/Admixture plot of 1,000 UAE nationals and 1,043 samples from the human genome diversity project (HGDP). From the phylogenetic tree of 1,000 genotype arrays, 120 UAE samples (predominantly cyan color with outline) were selected for whole genome sequencing. The admixture of UAE samples is shown as pie charts, with sector coloring consistent with HGDP population colors. The zoomed-in views display the genetic diversity of the UAE population with admixtures predominated by Middle Eastern, Central/South Asia (large zoomed inset), and Sub-Saharan Africa (small zoomed inset).

Sample selection was performed to maximize diversity according to the method in section “Selection of Unrelated Samples and Admixture Calculation.” Figure 2 shows our unique representation of diversity using principal component analysis (PCA) that incorporates admixture plot data as a pie chart used for selecting samples of interest for sequencing. The zoomed insets in Figure 2, clearly reflects the genetic diversity of the UAE population. Although around 40% of the samples have a major Middle Eastern admixture component (>70%), the rest of the samples shows significant admixture with other world populations predominantly with Central/South Asia and Sub-Saharan Africa.

The phylogenetic selection method (see sections “Materials and Methods” and “Selection of Unrelated Samples and Admixture Calculation”) included samples across the entire plane spanned by the first principal components (*X*-axis). Samples with different admixture proportions were selected. Specifically, samples with nearly 100% Middle-eastern component, as well as representatives with Sub-Saharan African and Central/South Asian components were selected. This shows that the selection method was capable of capturing the genetic diversity that was determined by the array-based genotype pre-analysis.

The mapping statistics for the 153 raw sequencing reads that were mapped to the hg19 reference genome were calculated and shown in Table 1. The quality of the mapping data affects the quality of the downstream processing of the data. The results were consistent with high-quality mapping scores with an average mapped read percentage of 87 and 99% and a mean mapping quality of 49 and 57 (with 60 being the highest possible score) for the WGS and WES, respectively. Furthermore, average coverages of 28.8X for the WGS and 44.9X for the WES were established.

**TABLE 1:** The average alignment and genome coverage for the 153 WGS/WES from mapping the raw reads of each sample to the hg19 reference genome.

Sequencing Type	Number of Samples	Number of reads	Mapped reads	Mapped reads %	Coverage X	Median insert size	GC content	Mean mapping quality
**Whole Genome Sequencing**	120	717,002,612	634,706,849	87.02	28.8	397	40.5	49
**Whole Exome Sequencing**	33	48,165,294	47,989,908	99.6	44.9	174	47.9	57

*The statistics reflect the quality of the mapping in terms of coverage, mapped reads percentages, and mean mapping quality.*

### Joint Genotyping and UAE Specific AF Calculation

Genotyping and variant calling of the 153 genomes of the UAE nationals resulted in 29,165,331 million variants of which 23,038,090 passed the GATK VQSR filter. Of the total number of variants, 92% (21,247,919) were “known” with respect to dbSNP (Sherry et al., 2001) (Build151) and 8% were “novel” (Table 2). The number of variants with major alternative allele frequencies (MAAF) was calculated by considering each AF from the 153 samples and then selecting those with AFs that were different from the reference by greater than 50%. The MAAF for each chromosome is shown in Table 2.

**TABLE 2:** The summary of autosomal and sex chromosomes’ variants called by genotyping of the 153 UAE nationals showing the known and novel number of variants based on their overlap with dbSNP (Build151).

	Chr	Integrated 153 UAE genomes								
		Variants	Known		Novel		Major alt allele			
			Number	%	Number	%	Number	%	Novel	%
**Autosomal Chr**	**All**	**23,038,090**	**21,247,919**	**92%**	**1,790,171**	**8%**	**2,067,743**	**9%**	5,371	0.3%
	**chr1**	1,753,942	1,614,772	92%	139,170	8%	159,936	9%	443	0.3%
	**chr2**	1,893,949	1,744,347	92%	149,602	8%	169,361	9%	401	0.2%
	**chr3**	1,605,197	1,482,800	92%	122,397	8%	141,490	9%	342	0.2%
	**chr4**	1,612,929	1,495,903	93%	117,026	7%	152,874	9%	323	0.2%
	**chr5**	1,441,391	1,332,308	92%	109,083	8%	123,409	9%	261	0.2%
	**chr6**	1,381,217	1,279,665	93%	101,552	7%	118,450	9%	286	0.2%
	**chr7**	1,292,726	1,192,113	92%	100,613	8%	111,779	9%	364	0.3%
	**chr8**	1,269,138	1,173,246	92%	95,892	8%	107,221	8%	218	0.2%
	**chr9**	954,499	881,164	92%	73,335	8%	83,312	9%	179	0.2%
	**chr10**	1,116,689	1,033,171	93%	83,518	7%	104,361	9%	248	0.2%
	**chr11**	1,090,783	1,005,373	92%	85,410	8%	109,961	10%	252	0.2%
	**chr12**	1,081,267	998,193	92%	83,074	8%	99,506	9%	253	0.3%
	**chr13**	805,263	745,938	93%	59,325	7%	84,011	10%	151	0.2%
	**chr14**	737,568	681,590	92%	55,978	8%	65,662	9%	185	0.3%
	**chr15**	642,562	593,597	92%	48,965	8%	59,752	9%	126	0.2%
	**chr16**	723,987	670,246	93%	53,741	7%	60,943	8%	124	0.2%
	**chr17**	624,153	573,102	92%	51,051	8%	53,909	9%	184	0.3%
	**chr18**	627,449	581,894	93%	45,555	7%	60,977	10%	127	0.2%
	**chr19**	520,807	479,141	92%	41,666	8%	42,096	8%	139	0.3%
	**chr20**	507,037	469,093	93%	37,944	7%	41,101	8%	74	0.2%
	**chr21**	312,621	290,169	93%	22,452	7%	31,427	10%	75	0.2%
	**chr22**	312,495	289,261	93%	23,234	7%	24,838	8%	83	0.3%
**Sex Chr**	**chrX**	715,067	631,707	88%	83,360	12%	60,389	8%	468	0.8%
	**chrY**	15,354	9,126	59%	6,228	41%	978	6%	65	6.6%

*The major alternative alleles are the variants with AF > 50% in the UAE samples.*

**FIGURE 3:**
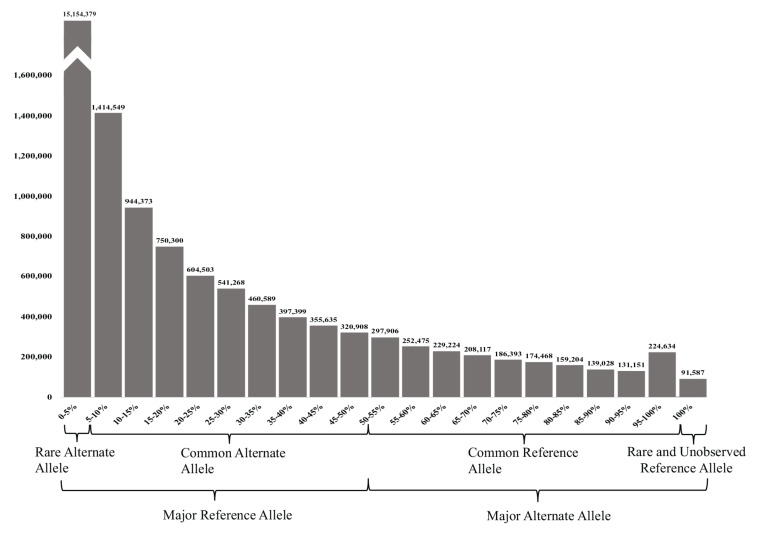
Allele frequency (AF) histogram of all filtered variants identified in the 153 samples. The histogram shows the number of variants against their AFs in (5% intervals). The highest peak represents the number of variants with rare alternate AFs of <5%, while the less common (lowest number of variants) had the rare or unobserved reference allele with 100% alternate AFs.

The 23,038,090 variants were categorized into two main groups (major reference allele and major alternate allele). These two groups were further subcategorized into four groups based on its AF: (1) the UAE rare alternate allele for those with an alternate AF of up to 5%, (2) common alternate allele (between 5 and 50% AF), (3) common reference allele (from 50 to 95% alternate AF), and (4) rare and unobserved reference allele (from 95 to 100% alternate AF) (Figure 3).

Predictably, the majority of the variants (65.78%) had a major reference allele with rare alternate AFs of <5, and 25.13% had the common alternate allele. Less common were the variants with MAAF, where 7.71% of the variants had a common reference allele of between 50–95 and 0.98% of the variants had a rare or unobserved reference allele with 95–100 and 0.40% had 100% alternate AFs (see Figure 3).

### United Arab Emirates Reference Genome Calculation and Testing

The UAERG was generated by integrating variants with MAAF frequencies of greater than 50% that were identified in the cohort of 153 UAE samples, into the hg19 reference genome. This resulted in the construction of the UAERG genome by replacing 2,067,743 sites in the hg19 reference genomes with the Major Alleles calculated for the UAE for both SNPs and INDELs.

The UAERG was subsequently tested using four genomes from samples that were not used to construct the reference genome. The variants for each of the four test samples were called by running the same variant calling pipeline twice using the two reference genomes hg19 and UAERG separately. Although no significant change in read per locus coverage was observed, the alignment quality improved with an increase of up to 5.3 million base pairs that were mapped to the UAERG in comparison to the hg19 reference. This, in turn, provided greater statistical power to variant calling where a reduction of up to 990,664 called variants per genome when using the UAERG in comparison to hg19 (see Table 3).

**TABLE 3:** Variant call reduction using the UAERG on the sequences from four UAE nationals.

		UAE S015				UAE S016				UAE S017				UAE S018			
	Chr	hg19	UAERG	Difference	Difference %	hg19	UAERG	Difference	Difference %	hg19	UAERG	Difference	Difference %	hg19	UAERG	Difference	Difference %
**Autosomal**	**chr1**	402,961	323,321	79,640	19.76	405,401	321,405	83,996	20.72	392,219	308,402	83,817	21.37	402,864	320,065	82,799	20.55
	**chr2**	412,000	324,350	87,650	21.27	403,261	318,001	85,260	21.14	399,028	313,695	85,333	21.39	412,509	329,532	82,977	20.12
	**chr3**	333,239	260,852	72,387	21.72	343,642	277,707	65,935	19.19	325,838	259,316	66,522	20.42	346,193	280,774	65,419	18.90
	**chr4**	358,300	281,472	76,828	21.44	364,340	287,789	76,551	21.01	354,895	278,450	76,445	21.54	379,594	306,839	72,755	19.17
	**chr5**	295,294	241,912	53,382	18.08	300,598	242,585	58,013	19.30	293,180	236,954	56,226	19.18	292,577	232,489	60,088	20.54
	**chr6**	286,974	232,909	54,065	18.84	300,306	242,939	57,367	19.10	280,917	225,918	54,999	19.58	289,538	233,502	56,036	19.35
	**chr7**	299,330	246,200	53,130	17.75	302,922	249,647	53,275	17.59	289,748	236,851	52,897	18.26	305,122	253,825	51,297	16.81
	**chr8**	257,291	204,661	52,630	20.46	258,232	206,143	52,089	20.17	252,234	200,180	52,054	20.64	252,235	202,257	49,978	19.81
	**chr9**	237,561	198,002	39,559	16.65	235,528	193,645	41,883	17.78	228,786	187,198	41,588	18.18	243,259	202,374	40,885	16.81
	**chr10**	251,860	200,937	50,923	20.22	261,363	211,786	49,577	18.97	245,953	192,623	53,330	21.68	257,948	202,587	55,361	21.46
	**chr11**	241,812	186,658	55,154	22.81	249,891	192,248	57,643	23.07	238,592	185,276	53,316	22.35	246,142	186,443	59,699	24.25
	**chr12**	231,836	180,522	51,314	22.13	241,123	193,887	47,236	19.59	225,556	175,733	49,823	22.09	233,523	183,316	50,207	21.50
	**chr13**	179,637	132,873	46,764	26.03	189,984	145,993	43,991	23.16	182,911	136,660	46,251	25.29	183,926	140,351	43,575	23.69
	**chr14**	158,917	127,299	31,618	19.90	169,846	135,970	33,876	19.95	163,253	129,121	34,132	20.91	168,611	135,653	32,958	19.55
	**chr15**	148,298	117,662	30,636	20.66	159,405	130,375	29,030	18.21	149,869	118,381	31,488	21.01	149,962	119,421	30,541	20.37
	**chr16**	166,647	137,621	29,026	17.42	166,194	135,931	30,263	18.21	164,002	134,748	29,254	17.84	171,105	140,772	30,333	17.73
	**chr17**	141,183	115,298	25,885	18.33	141,107	117,433	23,674	16.78	139,916	113,209	26,707	19.09	150,595	125,584	25,011	16.61
	**chr18**	141,259	108,956	32,303	22.87	139,704	108,268	31,436	22.50	137,584	105,299	32,285	23.47	144,134	111,593	32,541	22.58
	**chr19**	114,518	97,214	17,304	15.11	116,982	97,186	19,796	16.92	111,115	90,530	20,585	18.53	116,586	98,619	17,967	15.41
	**chr20**	114,760	96,896	17,864	15.57	120,655	102,872	17,783	14.74	116,883	97,734	19,149	16.38	113,170	94,965	18,205	16.09
	**chr21**	93,051	78,256	14,795	15.90	95,280	80,503	14,777	15.51	88,823	73,722	15,101	17.00	91,740	76,374	15,366	16.75
	**chr22**	70,197	59,724	10,473	14.92	72,992	61,902	11,090	15.19	71,356	61,994	9,362	13.12	76,564	65,426	11,138	14.55
	**Total/%**	**4,936,925**	**3,953,595**	**983,330**	**19.45**	**5,038,756**	**4,054,215**	**984,541**	**19.04**	**4,852,658**	**3,861,994**	**990,664**	**19.97**	**5,027,897**	**4,042,761**	**985,136**	**19.59**
**Sex**	**chrX**	109,924	78,173	31,751	28.88	155,003	127,580	27,423	17.69	153,143	124,112	29,031	18.96	105,958	77,079	28,879	27.26
	**chrY**	23,186	22,353	833	3.59	21,726	21,614	112	0.52	18,826	18,881	-55	-0.29	23,125	23,152	-27	-0.12
	**Total/%**	**133,110**	**100,526**	**32,584**	**16.24**	**176,729**	**149,194**	**27,535**	**9.10**	**171,969**	**142,993**	**28,976**	**9.33**	**129,083**	**100,231**	**28,852**	**22.35**

*The table shows the comparison of the variants called for each of the four test samples after running the pipeline twice using the two reference genomes hg19 and UAERG separately. The results were grouped into their respective chromosomes.*

### Comparison With Similar Data From the Region

A similar study conducted by Fakhro et al. (2016) compiled a catalog of around 24 million variants from 1,005 samples from the population of Qatar. For comparability purposes, the SNPs from this UAE effort were reduced to only contain biallelic data, resulting in 18,894,377 SNPs. When comparing the integrated (WGS and WES) autosomal biallelic SNPs from the UAE with the data from Qatar (20,159,695 SNPs), an overlap of 12,286,745 SNPs was found (Supplementary Figure S1).

While the overlap of 65% with the Qatari population was substantial, the distinct variants from the UAE population could be of added value to research efforts that seek to catalog global and local variants (Tadmouri et al., 2006; Scott et al., 2016). The frequencies of the UAE-specific variants were further analyzed. From the comparison with the Qatar dataset, 1,261,482 variants that occurred with an allele count (AC) >1 were unique to the UAE dataset. Moreover, in terms of AFs thresholds above 1, 5, 10, and 50%, 423,486; 23,974; 17,648; and 4,025 UAE specific variants were observed, respectively.

### Annotation of Variants

The resulting UAE database comprises 25,754,157 variants (19,929,600 SNPs, 2,827,383 short deletion, 2,906,803 short insertions, and 89,971 unknown). Additionally, all variants were annotated with AFs derived from this UAE study as well as AFs from gnomAD’s (Karczewski and Francioli, 2017) different populations. A total of 8,726,217 variants were found to be common in the UAE as they had AFs of more than 0.05, of which 611 variants were found to be UAE population-specific being rare (AF < 0.01) when compared to exons AFs from all gnomAD’s populations. When using genomes AFs instead of exons AFs from gnomAD, 13,112 UAE population-specific variants were identified. Moreover, the GEMINI (Paila et al., 2013) tool impact annotations were used to describe the functional consequence of the called variants. A total of 7,429, 98,288 and 25,645,131 variants were of high, medium, and low impact, respectively. Of greater interest, were the variants that were not found in repeat regions (using GEMINI Repeatmasker annotation) and were novel in contrast to dbSNP build 151 (Table 4).

**TABLE 4:** Characterization of variants by their GEMINI functional impact.

Impact severity	Variants impact	All	In repeat regions	In repeat regions “novel”	Not in repeat regions “novel”
High	Frameshift	3,403	516	198	1,468
High	Initiator codon	16	1	0	1
High	Splice acceptor	1,063	208	34	182
High	Splice donor	1,057	155	18	234
High	Start lost	231	9	4	26
High	Stop gained	1,537	113	38	426
High	Stop lost	122	12	2	19
Med	Disruptive inframe insertion	691	370	68	55
Med	Inframe deletion	643	301	37	52
Med	Inframe insertion	590	245	53	76
Med	Missense	94,941	3,849	661	10,378
Med	Disruptive inframe deletion	1,423	831	99	113
Low	3 prime UTR	223,737	49,374	5,076	16,416
Low	5 prime UTR premature start codon	7,215	853	74	627
Low	5 prime UTR	44,770	7,197	1,121	4,533
Low	Downstream gene	999,067	531,083	52,728	41,412
Low	Exon	40,623	14,979	1,326	2,030
Low	Intergenic	12,636,385	7,615,615	707,666	392,549
Low	Intragenic	23	13	2	0
Low	Intron	10,384,632	5,464,131	527,433	411,413
Low	Start retained	1	0	0	0
Low	Stop retained	272	4	0	4
Low	Synonymous	77,460	2,301	296	4,755
Low	Upstream gene	1,230,946	644,829	67,224	55,295

*The number of novel variants were listed to show those within and outside repeat regions using GEMINI’s RepeatMasker annotation.*

Table 5 shows the variants with high and medium functional effects that are specific to the UAE population. These were common in the UAE cohort (MAF > 0.05) but were rare or missing in all gnomAD populations (MAF < 0.01).

**TABLE 5:** United Arab Emirates specific variants with high and medium functional impact severity.

Impact severity	Chr	RS_id	Ref	Alt	Variant type	Functional impact	Gene symbol	Disease association (GeneCards)	UAE AF	gnomAD AF_all
High	1	rs765451626	CTG	C	Indel	Frameshift	SPEN	Breast liposarcoma; breast cancer; and brain cancer	0.05592	0.00334
	1	rs753994746	CAGCTT	C	Indel	Frameshift	ESPN	Deafness (autosomal recessive 36) with or without vestibular involvement and Usher syndrome type I	0.08609	0.00210
	2	rs527478913	TCGCA	T	Indel	Frameshift	NRP2	Wallerian degeneration; capillary hemangioma; and hirschsprung disease 1	0.05556	0.00391
	3	rs749453662	G	GTT	Indel	Frameshift	ZNF717	None	0.34545	0.00307
	11	rs368342230	TG	T	Indel	Frameshift	MUC6		0.05229	0.00000
	11	rs376177791	G	GT	Indel	Frameshift	MUC6	Pancreatic ductal carcinoma; tumor of exocrine pancreas; endocervical adenocarcinoma; signet ring cell adenocarcinoma; and gastric cancer	0.05882	0.00001
	11	rs780061827	AAT	A	Indel	Frameshift	MUC6		0.27124	0.00000
	11	rs769713098	G	GCA	Indel	Frameshift	MUC6		0.28431	0.00000
	19	rs770233746	GGCTT	G	Indel	Frameshift	MUC16	Clear cell adenocarcinoma and ovarian cyst	0.29934	0.00000
	X	rs1325813675	C	CTT	Indel	Splice acceptor	STAG2	Neurodevelopmental disorder; X-linked; with craniofacial abnormalities; and Xq25 duplication syndrome	0.16807	0.00298
	X	rs1325813675	C	CTTT	Indel	Splice acceptor	STAG2		0.12832	0.00327
	X	rs139484145	A	G	SNP	Stop lost	ARSD	Chondrodysplasia Punctata (tibia-metacarpal) and atrial septal defect 2	0.32895	0.00014
Medium	2	rs143372458	C	T	SNP	Missense	ANKRD23	Tibial muscular dystrophy; total anomalous pulmonary venous return 1; and dilated cardiomyopathy	0.05298	0.00464
	2	rs146511220	C	G	SNP	Missense	ACADL	Acyl-CoA dehydrogenase, very long-chain, and deficiency of acyl-CoA dehydrogenase deficiency	0.05556	0.00180
	2	rs141080282	G	A	SNP	Missense	LTBP1	Geleophysic dysplasia and brachydactyly, Type C	0.06250	0.00453
	2	rs142955097	A	G	SNP	Missense	WDR35	Short-rib thoracic dysplasia 7 with or without polydactyly and cranioectodermal dysplasia 2	0.07843	0.00148
	2	rs55660827	A	G	SNP	Missense	MCM6	Lactose intolerance, adult type, and lactose intolerance	0.17974	0.00027
	2	rs1400511133	GGGC	G	Indel	Disruptive inframe deletion	GDF7	Barrett esophagus	0.23077	None
	4	rs370593066	A	C	SNP	Missense	DCLK2	Lissencephaly; band heterotopia	0.07190	0.00085
	5	rs144066680	A	C	SNP	Missense	GPR151	Brachial plexus lesion	0.05229	0.00074
	7	rs1351676248	G	T	SNP	Missense	ZSCAN21	Retinitis pigmentosa 58	0.08553	0.00005
	7	rs75910050	G	A	SNP	Missense	FAM220A	Pancreatic squamous cell carcinoma	0.05882	0.00094
	11	rs675	T	C	SNP	Missense	APOA4	Carotenemia and demyelinating polyneuropathy	0.07292	
	16	rs149365469	C	T	SNP	Missense	ACD	Dyskeratosis congenital (autosomal dominant 6) and hoyeraal hreidarsson syndrome	0.05229	0.00075
	16	rs1799917	A	C	SNP	Missense	GNAO1	Epileptic encephalopathy, early infantile, 17, and neurodevelopmental disorder with involuntary movements	0.05556	0.00000
	17	rs140375987	C	T	SNP	Missense	CASC3	Bone marrow cancer; myeloma (multiple); chronic polyneuropathy; spherocytosis, Type 5; and Kabuki syndrome 1	0.05882	0.00111
	17	rs758821377	CTGT	C	Indel	Inframe deletion	KDM6B	Neurodevelopmental disorder with coarse facies and mild distal skeletal abnormalities; myelodysplastic syndrome; and kidney cancer; brain cancer	0.22000	0.00060
	19	rs8107444	A	T	SNP	Missense	ZNF28	None	0.07813	0.00004
	19	rs1427739410	GGGC	G	Indel	Inframe deletion	BTBD2	None	0.56818	None
	20	rs778174473	AGGGCCA GGGCCG	A	Indel	Disruptive inframe deletion	TAF4	Huntington disease	0.16667	0.00069
	X	rs78034736	G	T	SNP	Missense	ARSD	Chondrodysplasia punctata, tibia-metacarpal type; and atrial septal defect 2	0.30921	0.00006
	X	rs73632978	G	A	SNP	Missense	ARSD		0.32026	0.00014
	X	rs67272620	A	T	SNP	Missense	ARSD		0.32353	0.00008
	X	rs67359049	C	T	SNP	Missense	ARSD		0.32353	0.00007
	X	rs73632975	A	T	SNP	Missense	ARSD		0.32353	0.00004
	X	rs73632976	C	T	SNP	Missense	ARSD		0.32353	0.00006
	X	rs370769167	C	T	SNP	Missense	ARSD		0.32680	0.00001
	X	rs115332247	C	A	SNP	Missense	ARSD		0.32680	0.00002
	X	rs73632977	A	T	SNP	Missense	ARSD		0.32680	0.00012
	X	rs73632953	T	C	SNP	Missense	ARSD		0.32895	0.00015
	X	rs73632954	A	G	SNP	Missense	ARSD		0.32895	0.00002
	X	rs143238998	A	C	SNP	Missense	ARSD		0.32895	0.00001
	X	rs150899882	C	A	SNP	Missense	ARSD		0.32895	0.00001
	X	rs113556864	CCCAC GCCGG	C	Indel	Disruptive inframe deletion	ARSD		0.32895	0.00001

*The UAE allele frequencies were calculated from the variant calling of the 153 genome samples. These were compared with the genome AF from all populations in gnomAD. The table shows the variants that were common in UAE (MAF > 0.05) but were rare or missing in all gnomAD populations (using AFs from exonic regions).*

### Loss of Function Analysis

A total of 1,669 variants with an AF of >1% that cause a loss of function (LoF) in the genes according to GEMINI were identified. There were 1,033 indels, 625 SNPs, and 11 of unknown variant types. If we further narrow the search for LoF variants to those common in the UAE samples (AF > 5%) and rare elsewhere (i.e., less than 1% in all gnomAD populations when using AFs from the gnomAD exon catalog), 15 variants with AFs between 0.052 and 0.345 were detected. Four of the 11 affected genes were associated with different cancer types: MUC6 and ZNF717 (prostate), SPEN (breast), and STK33 (pancreas). While 3 other genes were associated with inborn genetic diseases: NRP2 (Hirschsprung disease 1), STAG (stag2 related disorder), and HTT (Huntington’s Chorea) (Supplementary Table S3).

### Heterozygosity/Homozygosity Ratios

The heterozygosity to homozygosity (het/hom) ratio is not only a tool for QC, it also provides an indication of ancestry (Wang et al., 2015). We found that UAE nationals with high Sub-Saharan African admixture also had a high het/hom ratio. Specifically, the individual (UAE_S120) with the highest Sub-Saharan African admixture (58.2%) had a het/hom ratio of 2.135 and was also the individual with the lowest inbreeding coefficient F_ST_, at 0.405. On the other hand, the sample (UAE_S116) with the lowest het/hom ratio (1.25) had an inbreeding coefficient of 0.562 (Supplementary Figures S2, S3) and has 99.9% Middle Eastern ancestry (Supplementary Table S1). Throughout the entire cohort, we observe a strong negative correlation between *F*_ST_ and Sub-Saharan African admixture, with Pearson coefficient of −0.745. Likewise, we observe a weak positive correlation between *F*_ST_ and Middle Eastern component with Pearson coefficient 0.406 (Supplementary Figure S3).

### Mitochondrial and Y-DNA Ancestry Haplogroups of the UAE Population

The distribution of the mtDNA haplogroups within the UAE population showed influences from populations in neighboring countries as well as from remote geographical regions. The haplogroup analysis revealed the major influence of H, J, K, T, and U. Fifty five percent of the study group carried these haplogroups that predominant in Europe and West Asia. The next most common haplogroups were the R and M (21%) haplogroups that are mainly found in Central/South Asia followed by the African haplogroups L at 17%. Finally, the N, G, D, and B haplogroups together were found in 7% of the study group, confirming a weaker influence from East Asia (Supplementary Figure 4a).

The analysis of the Y haplogroups in male subjects showed major influences from the Middle East and Central/South Asia. The most common J haplogroup was found in 52% of the study group, a lineage that is mainly found in the Middle East. The next largest group at 21% was the E haplogroup, primarily found in West and East Africa. Fourteen percent of the study group carried the R haplogroup that is primarily found in Central and South Asia as well as in Eastern Europe. There were minor influences from the G, L, T, and Q haplogroups at 5, 4, 2, and 1%, respectively, haplogroups that are native to Central South Asia and the Middle East (Supplementary Figure 4b).

### Structural Variants Call Set Generation and Evaluation

We generated SV call sets using the Manta (Chen et al., 2016) and Delly (Rausch et al., 2012) tools. The rationale and choice of integrating multiple SV-callers as an effective approach for SV discovery was based on previous analyses (Werling et al., 2018; Chaisson et al., 2019; Collins, 2019). This approach has been shown to provide a balanced sensitivity and specificity due to combining the benefits from the used SV calling tools. Specifically, Manta was selected for its leading performance among all paired-end/split reads algorithms and DELLY for its maximization of sensitivity for small and balanced SV.

**FIGURE 4:**
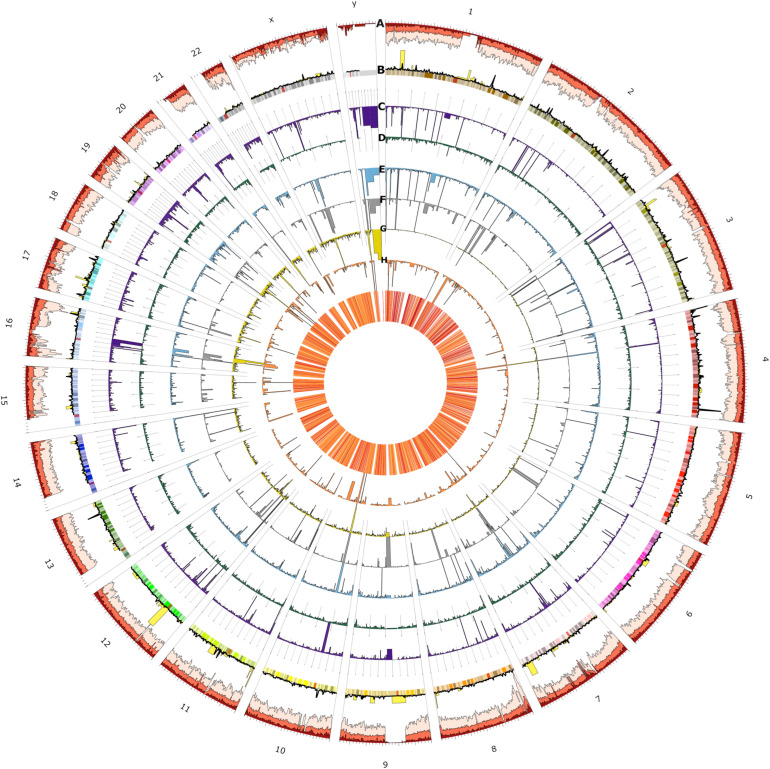
Circos plot of the spatial distribution of short variants and structural variants (SVs) across all chromosomes (outer ring). From outer to the inner rings: **(A)** short variants called from the 153 UAE samples (light red), novel (red), and novel UAE population-specific (dark red) variations which indicate regional variability characteristics (note that scales are modified for visibility), **(B)** loss of function (yellow) and UAE population specific variants (black line), **(C)** SVs consensus set (dark purple), of which the **(D)** through **(G)** rings show insertions (dark green), deletions (blue), duplications (gray), translocation (dark yellow), and inversions (orange), respectively. The heatmap in the innermost plot of the figure displays the frequency of SVs.

Depending on whether recall or precision was prioritized in subsequent analysis steps, results on both the intersection and union of the call sets were reported. For the union call set, 137,713 SVs (present in at least five individuals), including 8,845 inversions, 11,818 insertions, 27,620 duplications, 40,582 deletions, and 48,902 transitions (Supplementary Table S2) were identified. The AF distribution is shown in Supplementary Figure S5. A genome overview of the short and SVs sets using Circos (Krzywinski et al., 2009) is shown in Figure 4.

The figure shows spatial variability characteristics and allows for the identification of chromosomal positions with remarkable regional features. The variants at prominent peaks were used to perform Reactome Pathways (Croft et al., 2011) Enrichment analysis. The analysis showed that the peaks of the UAE population specific variation (black line, ring B) in chromosomes 1, 3, 4, and 13 corresponds to significant increase in variations in Glycosaminoglycan metabolism (*p*-value = 4.8 × 10^–5^), chondroitin sulfate/dermatan sulfate metabolism (*p*-value = 8 × 10^–5^), HS-GAG degradation (*p*-value = 6.05 × 10^–4^), Cristae formation (*p*-value = 0.001), Metabolism of carbohydrates (*p*-value = 0.001) and MPS I – Hurler syndrome (*p*-value = 0.002) pathways (Supplementary Figure S6). The LoF variations (yellow bars, ring B) peaks in chromosomes 1, at the end of 8, at the start of 11 and in 12 contained enriched variation in PTK6 promotes HIF1A stabilization (*p*-value = 0.031), signaling by high-kinase activity BRAF mutants (*p*-value = 0.016), transcriptional regulation of pluripotent stem cells (*p*-value = 0.016), and MAP2K and MAPK activation (*p*-value = 0.018) pathways (Supplementary Figure S7). Similarly, the peaks from the overall SV (purple bars, ring C) in chromosomes Y, 4, 10, 16, and 21 contained enriched variations that correspond with synthesis of PI (*p*-value = 0.004), TP53 regulated transcription of death receptors and ligands (*p*-value = 0.008), hemostasis (*p*-value = 0.009), and Cytosolic sulfonation of small molecules (*p*-value = 0.017) pathways (Supplementary Figure S8). In-depth analysis of these peaks could be of interest for further investigation when studying genomes from the UAE and those from populations of neighboring countries.

## Discussion

Using best practices described by Van der Auwera et al. (2013) for genome data QC, alignment, variant discovery techniques, and High-Performance Computing, the first UAERG was constructed. It was based on 153 high coverage samples from healthy subjects selected to represent the UAE nationals. This was achieved by using an in-house pipeline that mapped sequenced reads to the hg19 reference genome and then used the GATK4 (GVCF) genome analysis workflow (Figure 1) that in turn deployed joint genotype calling of the UAE genomes.

To avoid sampling bias, the WGS samples were carefully chosen from a cohort of 1,000 samples for which genotype array data was available. The choice of the representative samples was guided by a systematic phylogenetic analysis that selected samples from different parts of the tree to include samples with different ethnic admixtures that better represented the current UAE population.

The analysis of Y-DNA showed strong paternal influences from the Middle East and West Asia. The mtDNA analysis revealed a more diverse maternal origin with possible influences from North Africa, the Middle East; West, East, and South Asia as well as Europe, which could suggest that the diversity of the UAE population is influenced to a greater extent by the maternal ancestry.

The frequencies of all variants in the UAE genomes were calculated and their position relative to the NCBI reference genome hg19 were identified. Over 2 million alleles on hg19 were altered by replacing all the positions with the UAE major alternative alleles with frequencies of 50% or higher, a strategy that was used in the construction of the Qatar and Vietnam reference genomes (Thanh et al., 2015; Fakhro et al., 2016). From this effort, a reference genome for the UAE was generated for the first time. The UAERG should be more compatible with UAE genomes than the reference genomes that have been widely used to date. The UAERG is expected to be a fundamental tool that will advance genome and public health research in the UAE.

The benefit of the new reference genome was established by testing four WGS from UAE individuals by showing significant enhancement of the genotype calling quality in the form of a reduction of approximately 19% called variants which corresponds to approximately one million less called variants.

The UAE-specific AFs will decrease the rates of misdiagnosis of genetic diseases that mainly depend on values from populations with genetic compositions that differ from the UAE and that, for example, by ruling out known disease-causing variants with high AFs in the UAE population. The variant reduction; in the magnitude of hundreds of thousands due to the use of the UAERG; is deemed helpful in the variant filtration step when searching for disease causing variants by reducing the number of variants that need to be considered in the analysis. Furthermore, records of variants with MAAF > 50% were cataloged as well as their associated pathogenicity whenever available (Supplementary Table S4).

Although the use of data from similar populations in the region could be a beneficial resource, as one could see in the comparison with the Qatari variants (65% of the generated UAE variants were overlapping with the Qatari data), there was still a significant difference (more than 6 million) variants between the two populations. These results show, that the diversity of the Arabian Peninsula has not been exhaustively covered by previous studies and that our dataset provides a substantial contribution toward that goal.

Furthermore, this reference panel is an inevitable component for high accuracy genotype imputation, which enriches datasets from cost efficient genome sequences from SNP arrays.

The sequencing was based on short-read technology, which limits SV discovery as well as *de novo* assembly. However, as demonstrated by Collins (2019), the use of various SV callers can significantly reduce the number of undetected variants.

In addition, the integration of population specific variants into a reference genome avoids coordinate lift-over inaccuracies derived from *de novo* assemblies, which remain a challenge for subsequent annotation procedures. Moreover, the homogeneous quality of samples enables the extraction of population characteristics from a population more evenly, in contrast, to study designs with emphasis on the high quality of a single or few sample(s). Therefore, samples with low coverage are likely to contribute less to a consensus genome due to their smaller call sets. As it is unlikely that quality/coverage reflects representativeness, we here avoid this study-based representation bias.

## Data Availability Statement

The VCF file including the variants and allele frequencies produced by this study is available at the European Genome Archive (EGA) under the accession number “EGAS00001004537”.

## Ethics Statement

The studies involving human participants were reviewed and approved by the Institutional Ethics Committee of Mafraq Hospital in Abu Dhabi (MAF-REC_07). The patients/participants provided their written informed consent to participate in this study.

## Author Contributions

HA and GT conceived the 1,000 UAE genome project and received funding that contributed toward sequencing the individuals in this publication. GD and AH performed the analyses and prepared the first draft of the manuscript. All authors developed the manuscript further and contributed to the final version.

## Conflict of Interest

The authors declare that the research was conducted in the absence of any commercial or financial relationships that could be construed as a potential conflict of interest.
