# Supplementary material for: A Population-Specific Major Allele Reference Genome From The United Arab Emirates Population
Source: Front Genet. 2021 Apr 23;12:660428. doi: 10.3389/fgene.2021.660428 (PMC8102833; doi:10.3389/fgene.2021.660428)
Supplement: Supplementary file 1 [file Data_Sheet_1.docx]

A Population-Speciﬁc Major Allele Reference

Genome From The United Arab Emirates Population.

Gihan Daw Elbait^1†^, Andreas Henschel^1,2†^, Guan K. Tay ^1,3,4,5^ and Habiba S. Al Safar ^1,3,6*^

^1^ Center for Biotechnology, Khalifa University of Science and Technology, Abu Dhabi, United Arab Emirates,

^2^ Department of Electrical Engineering and Computer Science, Khalifa University of Science and Technology, Abu Dhabi, United Arab Emirates, ^3^ Department of Biomedical Engineering, Khalifa University of Science and Technology, Abu Dhabi, United Arab Emirates, ^4^ Division of Psychiatry, Faculty of Health and Medical Sciences, The University of Western Australia, Crawley, WA, Australia, ^5^ School of Medical and Health Sciences, Edith Cowan University, Joondalup, WA, Australia, ^6^ Department of Genetics and Molecular Biology, College of Medicine and Health Sciences, Khalifa University of Science and Technology, Abu Dhabi, United Arab Emirates.

Supplementary Tables and Figures


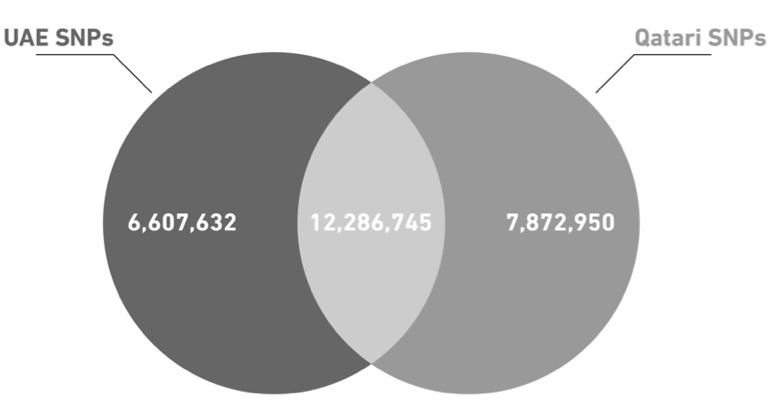


Figure S1: Venn diagram showing an overlap of 12,286,745 SNPs in a comparison between the UAE and the Qatari population sequencing study.

Table S1: The UAE Samples with their corresponding admixture proportions values from the eight HGDP populations.


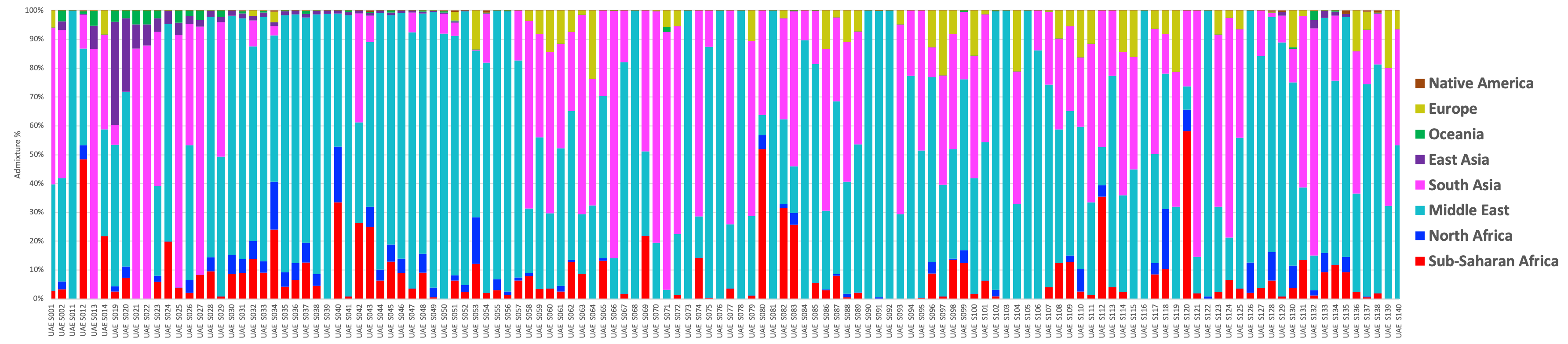

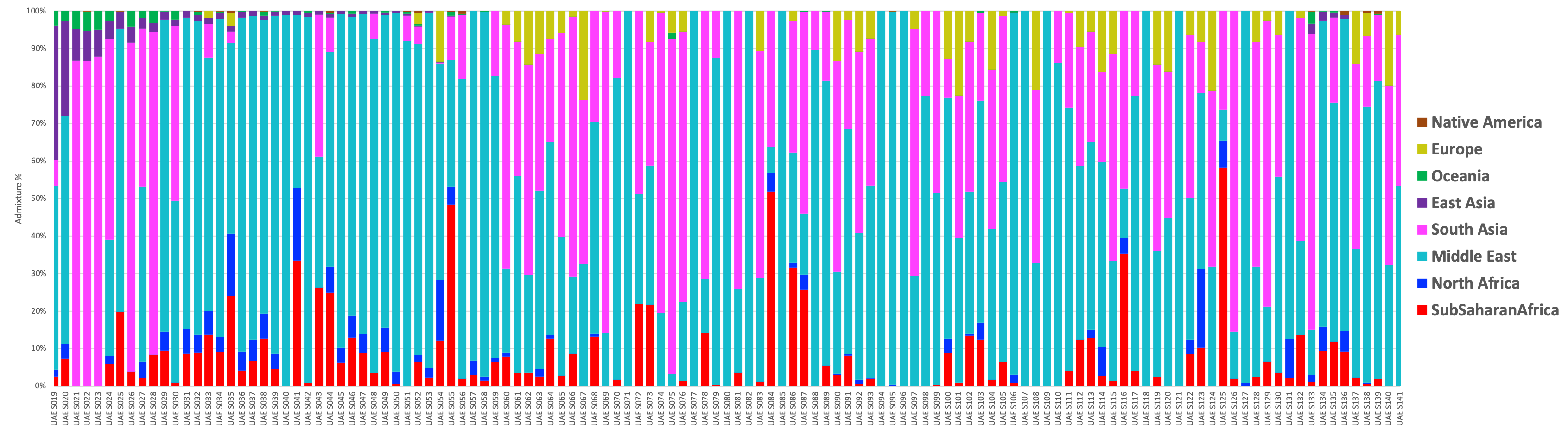


Figure S2: The selected UAE Samples with their corresponding admixture proportions colored according to the eight HGDP population.


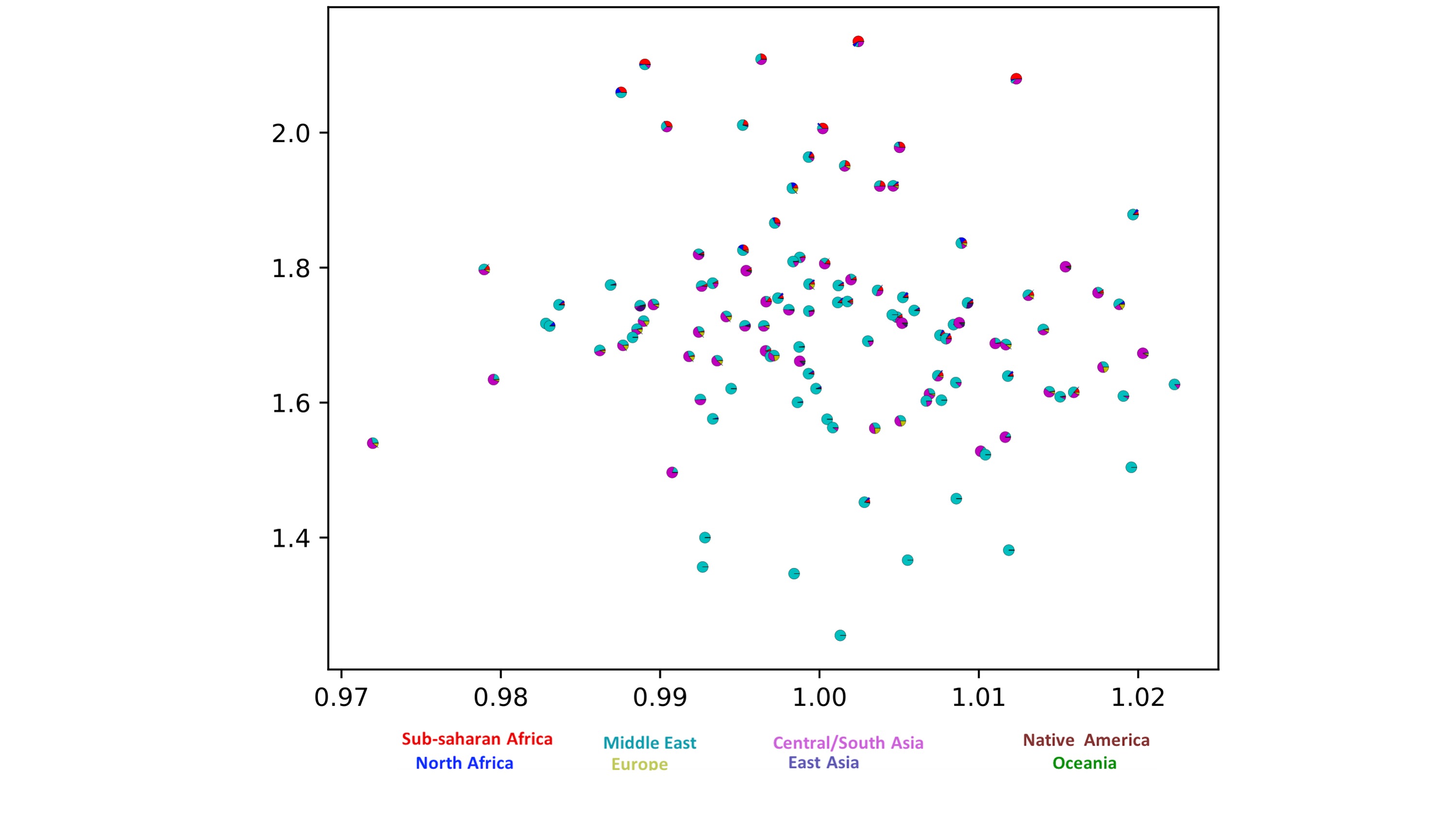


Figure S3: Heterozygous to Homozygous(het/hom) ratio of all called variants for the WGS data. Each sample is represented with a pie chart reflecting its admixture ratios from eight world populations. The samples with higher het/hom ratio had a significant Sub-saharan African admixture and the samples with Lower het/hom ratio are mainly those of the Middle eastern ethnic group.

| Variants | All | 25,753,757 |
| --- | --- | --- |
| short | SNPS | 19,929,600 |
|  | Deletions | 2,827,383 |
|  | Insertions | 2,906,803 |
|  | unknown | 89,971 |
|  | Common | 8,726,217 |
|  | UAE specific | 611(Exonic) |
|  | UAE specific | 13,112 (whole genome) |
| Structural | All | 137,713 |
|  | Deletions | 40,582 |
|  | Insertions | 11,818 |
|  | Inversions | 8,845 |
|  | Duplications | 27,620 |
|  | Transitions | 48,902 |

Table S2: Called Short and Structural variants from the UAE samples.

Table S3: LoF variants to those common in our samples (AF>5%) and rare elsewhere (less than 1%) in all GnomAD populations.

| a)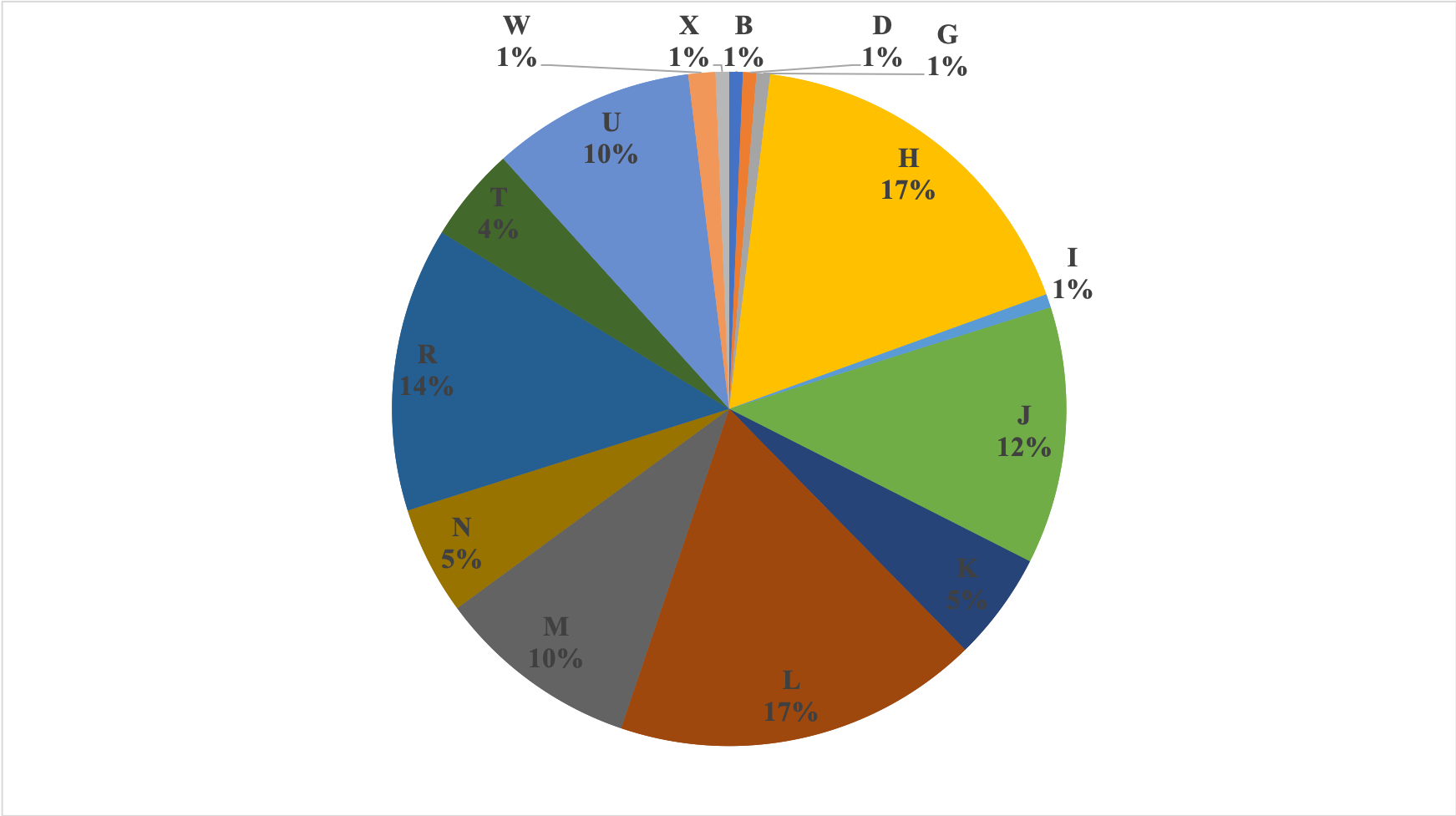 | b)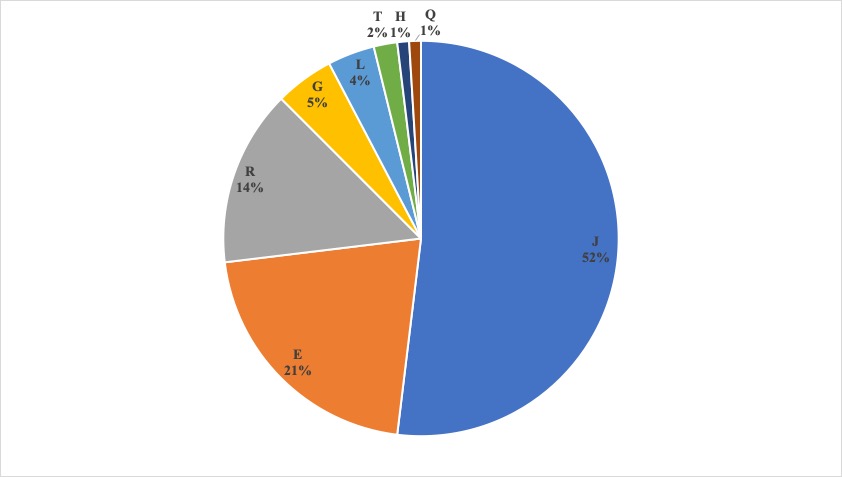 |
| --- | --- |

Figure S4: The UAE genomes: a) mtDNA and b) Y haplogroups distributions.


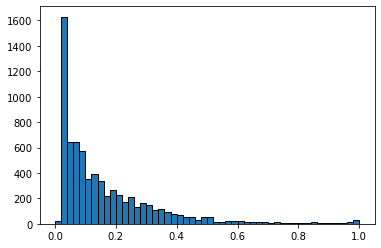


Figure S5: The Structural Variants Allele Frequency distribution generated from the UAE samples.

Table S4: The clinical annotation for variants using ClinVar "Pathogenic, Likely_Pathogenic " clinical significance with AFs generated from the 153 UAE samples.


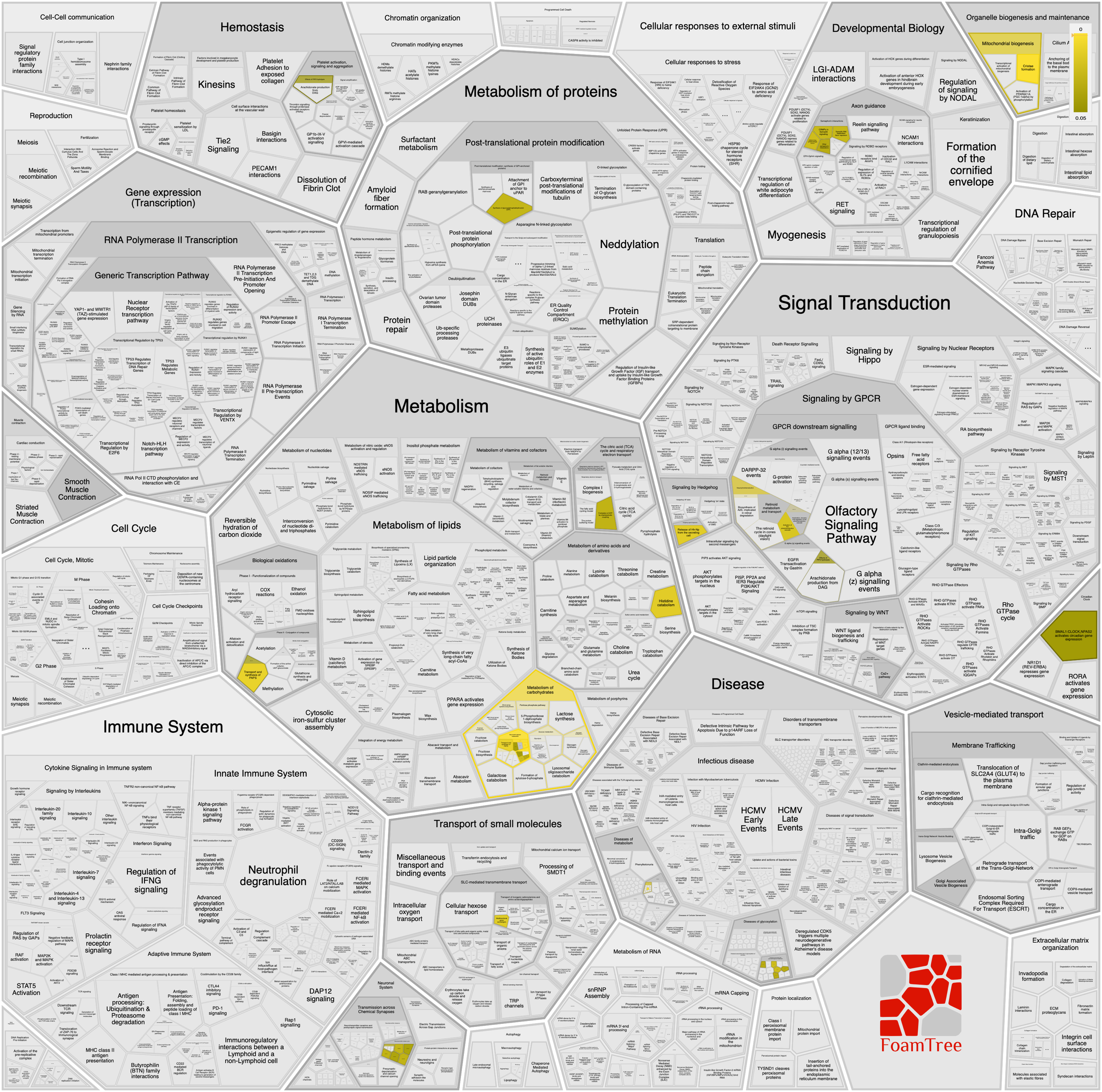


Figure S6: Reactome Pathways Enrichment Graph for UAE specific variants from selected prominent peaks in chromosomes 1, 3, 4, and 13.


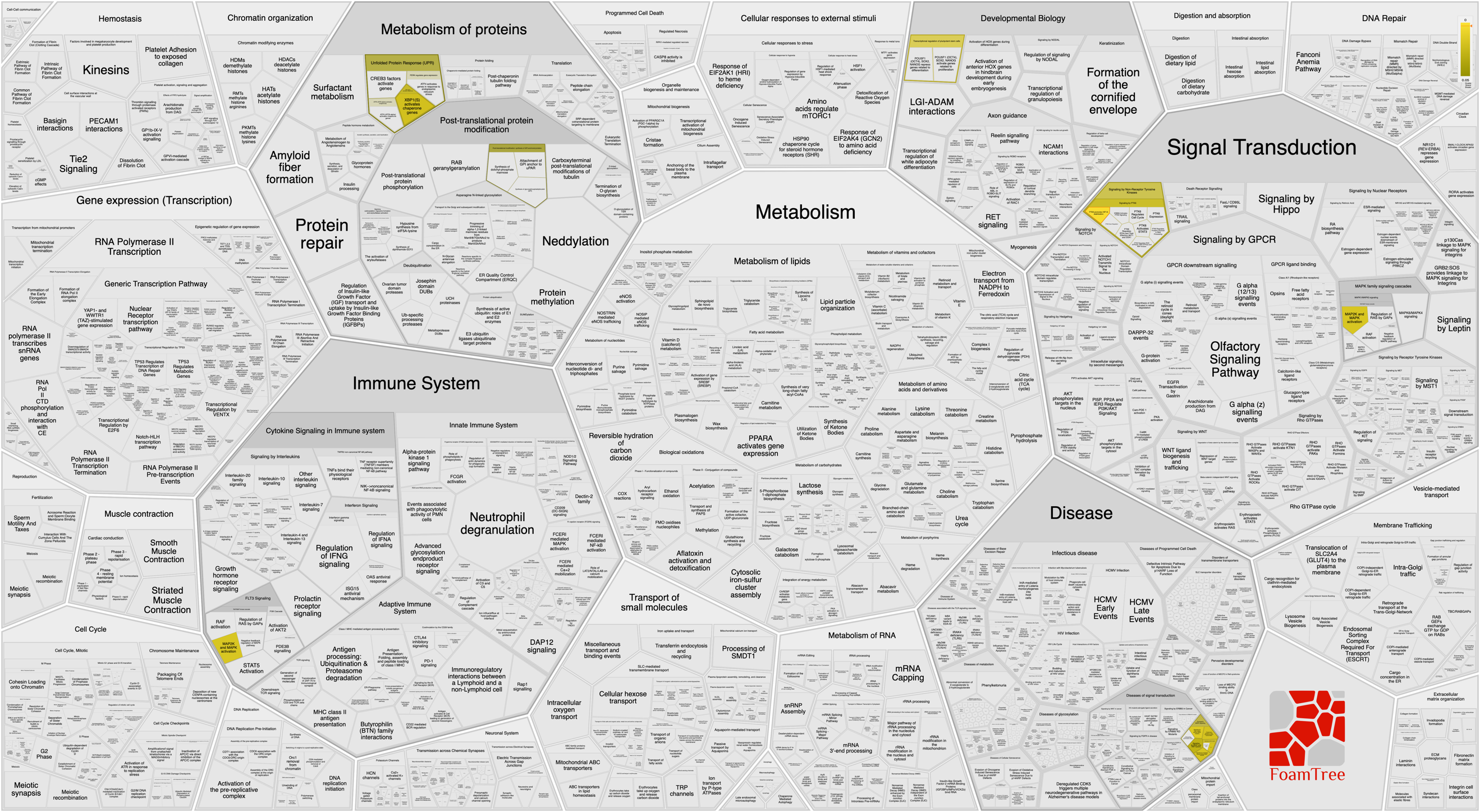


Figure S7: Reactome Pathways Enrichment Graph for LoF variants from selected prominent peaks in chromosomes 1, 8, 11, and 12


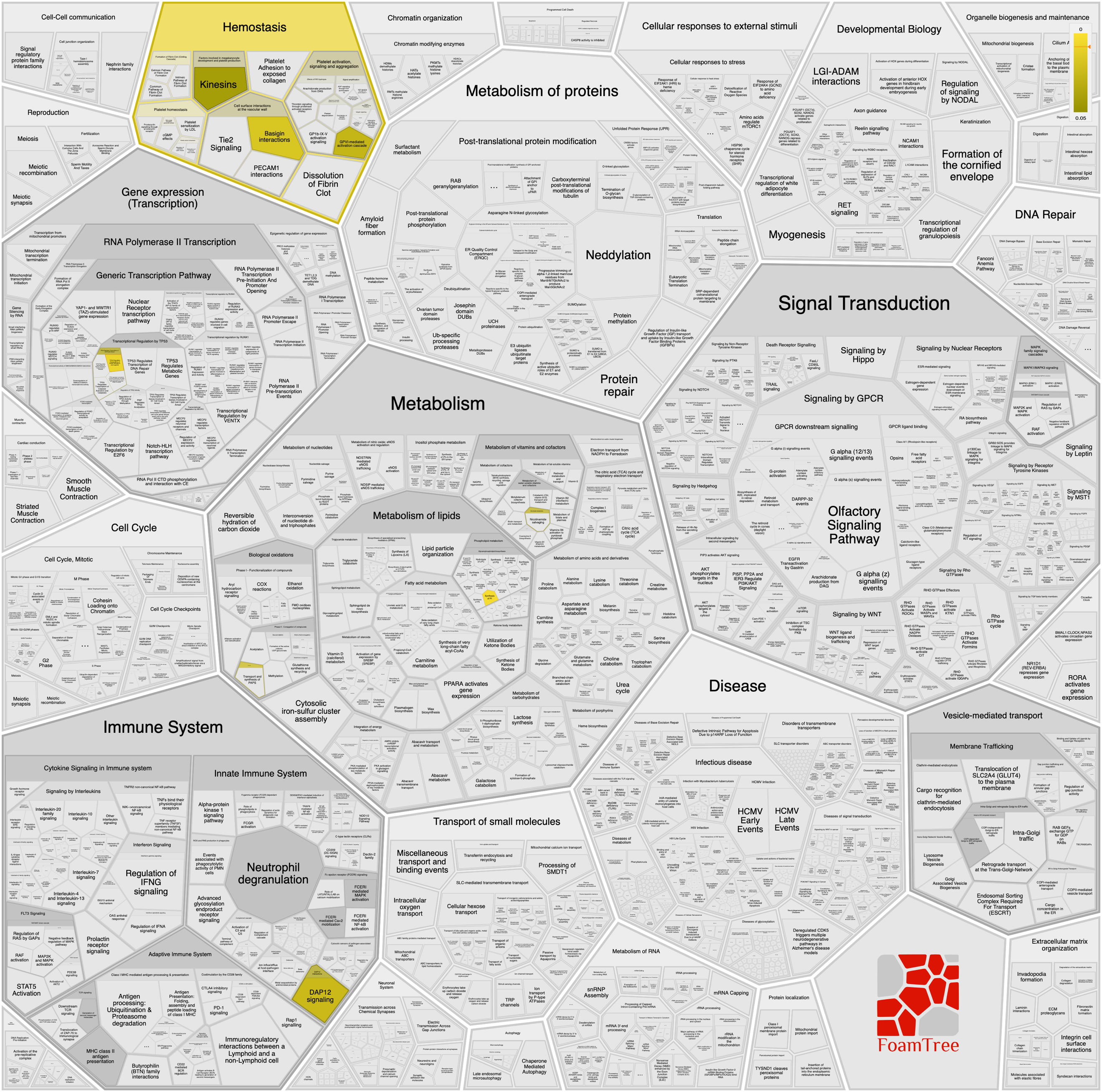


Figure S8: Reactome Pathways Enrichment Graph for Structural Variants from selected prominent peaks in chromosomes Y,4,10, 16, and 21.
